# Vinpocetine is the forthcoming adjuvant agent in the management of COVID-19

**DOI:** 10.2144/fsoa-2021-0099

**Published:** 2022-04-14

**Authors:** Hayder M Al-kuraishy, Ali I Al-Gareeb, Muyiwa Samuel Fageyinbo, Gaber El-Saber Batiha

**Affiliations:** ^1^Department of Clinical Pharmacology & Therapeutics medicine, College of Medicine, AL-Mustansiriyiah University, Baghdad, Iraq; ^2^Department of Pharmacology & Therapeutics, Faculty of Basic Clinical Sciences, University of Medical Sciences, Ondo City, Ondo State, PMB 536, Nigeria; ^3^Department of Pharmacology & Therapeutics, Faculty of Veterinary Medicine, Damanhour University, Damanhour, Albeheira, 22511, Egypt

**Keywords:** anti-inflammatory, anti-oxidant effects, COVID-19, hyper-inflammation, oxidative stress, phosphodiesterase inhibitor, pro-inflammatory cytokines, vinpocetine

## Abstract

Vinpocetine (VPN) is an alkaloid derivative of vincamine inhibits phosphodiesterase type 1 that increase cyclic guanosine monophosphate and cyclic adenosine monophosphate. VPN have anti-inflammatory and antioxidant effects with suppression release of pro-inflammatory cytokines. Moreover, VPN mitigates oxidative stress (OS) and inflammatory reactions through inhibition of mitogen-activated protein kinase (MAPK) signaling pathway. Therefore, VPN may decrease hyper-inflammation-induced acute lung injury in COVID-19 through modulation of NF-κB pathway. Taken together, VPN has pulmonary and extra-pulmonary protective effects against COVID-19 through mitigation of OS and hyperinflammation. In conclusion, VPN has noteworthy anti-inflammatory and anti-oxidant effects through inhibition of NF-κB/MAPK signaling pathway so, it may reduce SARS-CoV-2-induced hyper inflammatory and OS.

## Plain language summary

Vinpocetine (VPN) is an alkaloid from vincamine with a potent anti-inflammatory and antioxidant effects. VPN has the possibility of inhibiting substances that cause inflammation and oxidative stress and as a result, may be beneficial in COVID-19 treatment. Due to the anti-inflammatory effect of VPN, acute lung injury in COVID-19 may reduce as a result of the inhibition of agent that causes lungs inflammation in COVID-19. VPN may have potential to reduce difficulty in breathing and damage to the lungs. Conclusively, VPN when administered with other therapy may improve the outcome of treatment of COVID-19.

## Graphical abstract

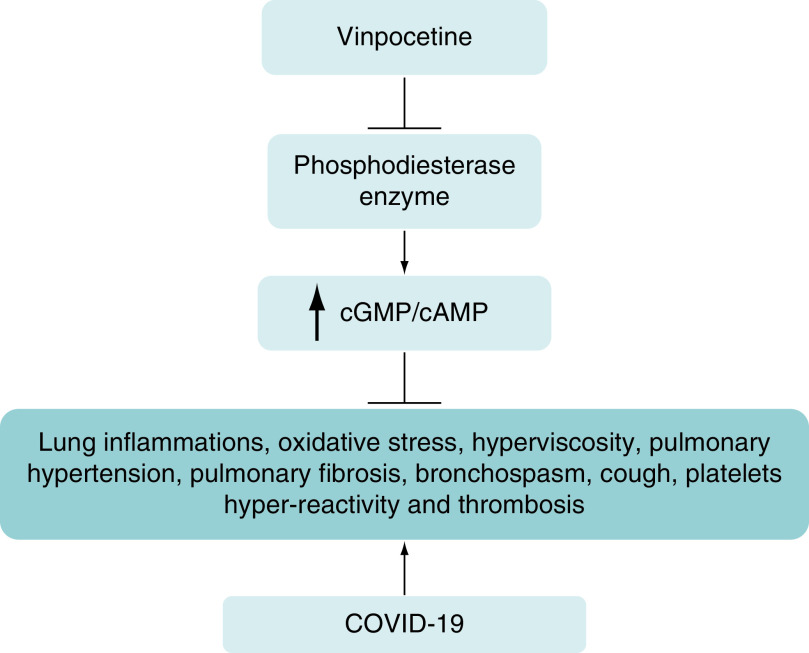

Vinpocetine (VPN) is an alkaloid derivative of vincamine with specific and unique chemical structure (Figure 1). VPN is used as dietary supplement to advance cognitive dysfunction and cerebrovascular complications associated with aging [1]. VPN is a phosphodiesterase (PDE) inhibitor, mainly type 1, inhibits both basal and activated PDE1, increase cyclic guanosine monophosphate (cGMP) and cyclic adenosine monophosphate (cAMP) [2]. Diverse types of PDEs exhibit different affinity for cGMP and cAMP. The PDE4, PDE7 and PDE8 isoforms hydrolyze cAMP, while PDE5, PDE6, PDE9 isoforms hydrolyze cGMP, though other isoforms have dual substrate specificities [2]. Schermuly *et al.* [3], showed that PDE1 was upregulated in the lung and pulmonary vessels in experimental pulmonary hypertension.

**Figure 1.**
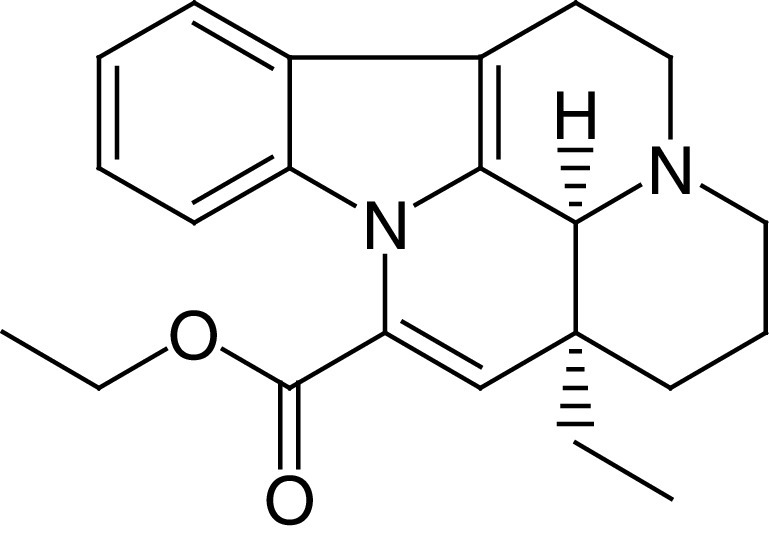
Chemical structure of vinpocetine.

In addition, VPN has anti-inflammatory effects through suppression the activity of NF-κB signaling pathway and release of pro-inflammatory cytokines such as IL-s, IL-8, IL-6, IL-1β and TNF-α [4]. Therefore, VPN restrain NF-κB-dependent pro-inflammatory cytokines release that is independent of PDE1 inhibition; thereby, the VPN anti-inflammatory effect is chiefly mediated via modulation of inducer-κB kinase (IKK) and degradation of IκB that regulate activity of NF-κB [5].

COVID-19 hypercytokinemia- and cytokine storm (CS)-induced multi-organ failure may occur in critical cases because of stimulation of NF-κB pathway [6]. Given that, high circulating IL-6 is linked with progress of acute respiratory distress syndrome (ARDS) [7]. Kircheis *et al.* [8], revealed that NF-κB signaling pathway is regarded as a main inflammatory signaling during COVID-19-induced release of pro-inflammatory cytokines and chemokines. The interaction between SARS-CoV-2 and angiotensin converting enzyme 2 (ACE2) provokes a higher inflammatory response in the lung and development of ARDS through activation of NF-κB pathway [9].

Moreover, different studies illustrated that NF-κB pathway inhibitors such as acetylsalicylic acid, indomethacin and dexamethasone suppress excessive immune stimulation and CS-induced-multi-organ failure [10]. Thus, VPN may have a great role in the management of COVID-19 through suppression of NF-κB and associated ALI. Yongshun *et al.* [11], demonstrated that VPN mitigates lipopolysaccharide induced-ALI in rats. Lugnier *et al.* [12], illustrated those PDEs mainly PDE4 are upregulated in both COVID-19 patients and chronic nicotine cigarette smoking, and linked with COVID-19 severity through upregulation of pulmonary ACE2. Therefore, VPN may decrease hyper-inflammation-induced ALI in COVID-19 through modulation of NF-κB pathway and PDE activity.

Indeed, SARS-CoV-2 directly activates various intracellular proteins such as nod-like receptor pyrin 3 (NLRP3) inflammasome, which through caspase-1 triggers liberation of pro-inflammatory cytokines [13,14]. It has been shown that SARS-CoV-2 may directly activate NLRP3 inflammasome through viroporin protein 3a and damage associated molecular patterns causing production release of pro-inflammatory cytokines [15]. NLRP3 inflammasome inhibitors such as tranilast, tetracycline, resveratrol, nicardipine, erythropoietin and colchicine are under clinical trials for management of COVID-19 [16]. It has been proposed that VPN inhibits NLRP3 inflammasomes induced-pro-inflammatory cytokines in the experimental rats [17]. As well, a recent study illustrated that VPN alleviates acute ischemic stroke associated inflammatory reactions through suppression of NLRP3 inflammasomes in mice [18]. Hence, VPN may mitigate COVID-19 severity and clinical outcomes through modulation of NLRP3 inflammasomes dependent immune overstimulation.

Notably, in severe SARS-CoV-2 infection, production of free radicals is increased, resulting in oxidative stress (OS) that induce local and systemic tissue damage [19]. Overwhelming OS induces the neutrophils into formation of neutrophil extracellular traps (NETs), and inhibits T cells that are essential to eradicate virus-infected cells. Therefore, SARS-CoV-2-induced OS may inhibit innate immune response [20]. Thus, different antioxidants like vitamin C and vitamin E may reduce the severity of COVID-19 [21]. In addition, high reactive oxygen species (ROS) leads to dysfunction of red blood cells, thrombosis, microvascular injury, disturbance of iron homeostasis, oxygen transport and tissue damage that are linked severity of COVID-19 [22]. VPN has noteworthy antioxidant effect via inhibition of macrophages and neutrophil superoxide anion production [23]. Also, VPN improves lipid peroxidation and endogenous antioxidant capacity with subsequent reduction of OS in the experimental acute kidney injury [18]. For these reasons, VPN may reduce SARS-CoV-2 infection-associated OS and related complications such as acute kidney injury in COVID-19.

Into the bargain, downregulation of ACE2 during SARS-CoV-2 infection, decrease level of vasodilator angiotensin (Ang)I–VII with augmentation of AngII [24]. High circulating AngII induces a sequence of inflammatory changes through stimulation of mitogen-activated protein kinase (MAPK) [25]. It has been illustrated that MAPK signaling pathway is implicated in SARS-CoV-2 pathogenesis through release of pro-inflammatory cytokines and induction of ALI/ARDS in severely affected COVID-19 patients, thus MAPK signaling pathway inhibitors may attenuate SARS-CoV-2-induced complications [26]. Different p38 MAPK inhibitors such as losmapimod and dilmapimod reduced ALI in various clinical trials [27,28]. Overactivation of p38 MAPK signaling pathway in the endothelium leads to platelet activation, thrombosis, endothelial cell apoptosis and cardiomyocyte injury [29]. Similarly, high p38 MAPK activity in lung causes pulmonary vasoconstriction and ALI [30]. Wang *et al.* [31], illustrated that VPN mitigates OS and inflammatory reactions through inhibition of p38 MAPK signaling pathway in diabetic rats. Moreover, Lee *et al.* [32], showed that VPN inhibits bacterial infection of respiratory mucosal epithelium through suppression of MAPK/ERK signaling cascade. Thus, VPN may prevent different secondary bacterial infections in critically ill COVID-19 patients.

Besides, high circulating AngII triggers expression of pulmonary PDE with subsequent inflammatory changes in SARS-CoV-2 infection [33]. Therefore, PDEIs like sildenafil had been successfully developed for management of pulmonary inflammation-induced hypertension in COVID-19. Indeed, tadalafil and sildenafil also suppress the transition of smooth muscles and endothelial to mesenchymal cells in the pulmonary vessels, preventing thrombotic and clotting complications [34]. As well, losartan, which is angiotensin receptor type 1 blocker is also regarded as PDE4 inhibitor, has important role in mitigation of lung inflammation storm with possible role in treating COVID-19 [35].

Of interest, COVID-19 is associated with various neurological manifestations including; confusion, headache, seizure and cognitive impairment due to direct SARS-CoV-2 brain tropism, endothelial dysfunction, cerebral thrombosis and systemic inflammatory disorders [36]. Golovacheva *et al.*, revealed that VPN has important role in the management of cognitive impairment in COVID-19 patients through regulation of brain neurotransmitters with noteworthy anti-inflammatory and anti-oxidant effects [37]. Besides, VPN and other nootropic agents may ameliorate SARS-CoV-2 infection-induced short and long-term neuropsychiatric disorders [38].

Therefore, in addition to the pulmo-protective effect of VPN, it has neuroprotective effects against neuropsychiatric disorders, so VPN could have dual central and peripheral protective effects in COVID-19 patients.

Of note, mutation in cardiac voltage-gated sodium channel (VGSC), which encoded by SCN5A gene may lead to sudden cardiac death due to arrhythmias in the Brugada syndrome [39]. High fever in COVID-19 induces overactivity of cardiac VGSC in patients with Brugada syndrome leading to electrical storm, which presented as ventricular arrhythmia and non specific ST-changes [39]. This syndrome is commonly treated by hypothermic protocol and quinidine, a potent anti-arrhythmic drug inhibits propagation of ventricular arrhythmia [40]. Zhang and colleagues reported that VPN inhibits cardiac VGSC, AngII-induced cardiomyopathic hypertrophic growth, cardiac fibroblast activation and suppression expression of fibronectin and matrix genes [41]. Thus, VPN has a cardioprotective effect against cardiomyocyte injury and arrhythmias, which are commonly linked with COVID-19-induced acute cardiac injury [42]. Therefore, ultimate effects of VPN are correlated with the suppression of PDE1, inducer-κB kinase and voltage gated sodium channel that involved in cell toxicity and death (Figure 2) [43].

**Figure 2.**
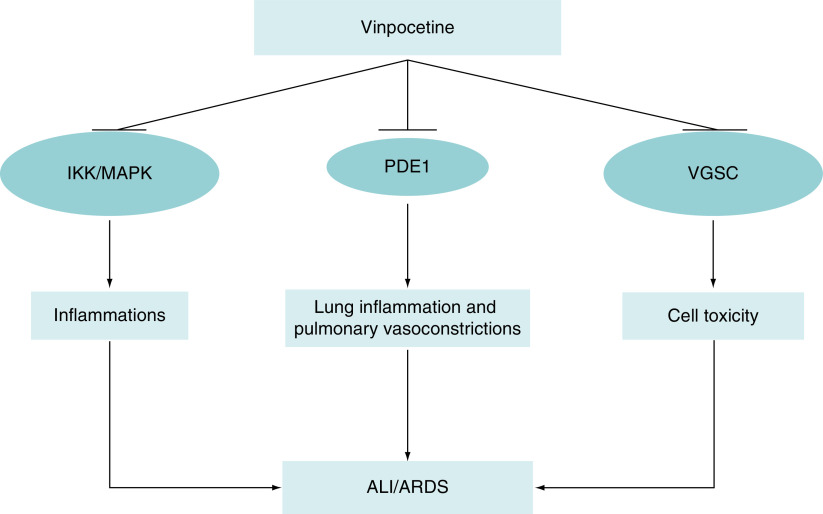
The potential mechanism of vinpocetine in the attenuation of acute lung injury and acute respiratory distress syndrome. Vinpocetine inhibits IKK, MAPK, PDE1 and VGSC-induced cell toxicity. Therefore, vinpocetine reduces risk of ALI and ARDS. ALI: Acute lung injury; ARDS: Acute respiratory distress syndrome; IKK: Inducer-κB kinase; MAPK: Mitogen-activated protein kinase; PDE1: Phosphodiesterase 1; VGSC: Voltage-gated sodium channel.

Moreover, VPN has significant antiplatelet and antithrombotic effects following single dose in patients atherosclerosis and ischemic heart disease through inhibition of von Willebrand factor and arachidonic acid-induced platelet aggregations with increases of erythrocyte deformability and improvement of endothelial dysfunction [44]. Therefore, VPN can be used as an adjuvant treatment to overcome aspirin resistance in patient with high risk of coagulopathy, since it reduces intra-plaque hemorrhage with increasing of atherosclerotic plaque stability [45].

It has been shown that in both influenza and SARS-CoV-2 infections, the platelets are activated by viral viroporins through TLR7 with subsequent platelets hyper-reactivity and risk of thrombosis [46]. The activated platelets together with neutrophils through P-selectin and activated complements contribute in formation NETs and release of thrombogenic DNA histone [47]. Thus, VPN through inhibition of platelets activity could reduce their interactions with the neutrophils and complements with attenuation of thrombogenic NETs formation. Besides, blood viscosity in COVID-19 is augmented due to erythrocyte deformity (ED), hyperinflammation and dehydration due to nausea and vomiting [48]. As well, mechanical stress caused by ED and atherosclerosis increase Ca^+2^ entries into the erythrocytes leading to decrease of erythrocyte deformability with subsequent high blood viscosity [48]. It has been documented that VPN inhibits Na^+2^-Ca^+2^ dependent pathways, through which enhance erythrocyte membrane deformability with reduction of blood viscosity [49]. What’s more, VPN exerts a vasorelaxant effect by activating release of nitric oxide (NO), which induces generation of cGMP. Therefore, VPN can attenuates tolerance to the nitroglycerine effect, which caused by over-expression of PDE1A [50]. Similarly, VPN inhibits AngII inhibitory effect on atrial natriuretic peptide-induced cGMP accumulation caused by over expression of PDE1A [50]. So, inhibition PDE1A by VPN may improve transpulmonary cGMP and pulmonary vasodilatation and prevent development of pulmonary hypertension [43]. Therefore, VPN might be of great value in attenuation of COVID-19-induced hyperviscosity, platelets-mediated microthrombosis and pulmonary complications.

Taken together, VPN has pulmonary and extra-pulmonary protective effects against COVID-19 through mitigation of OS, hyperinflammation, hyperviscosity, platelets hyper-reactivity and thrombosis (Figure 3).

**Figure 3.**
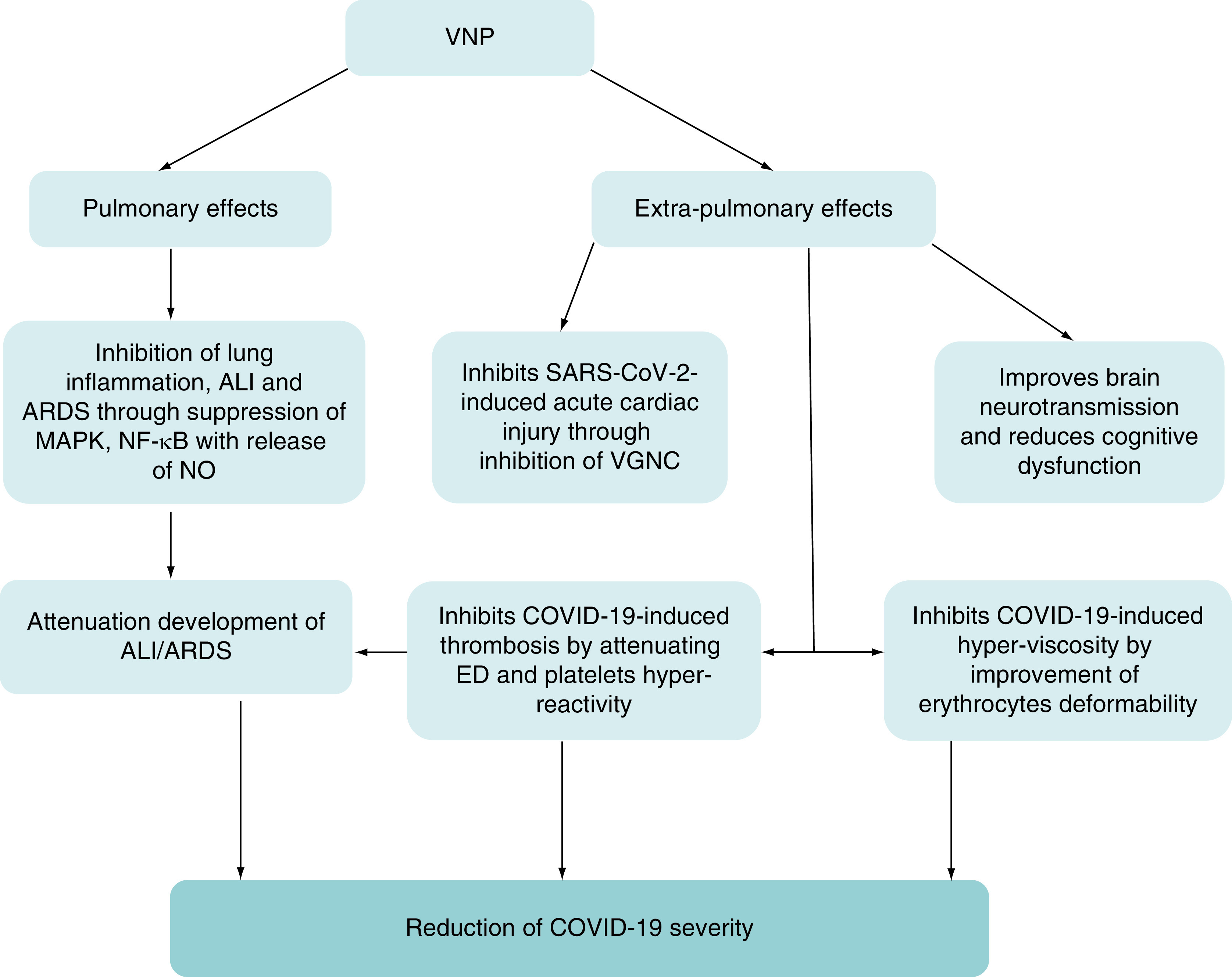
The pulmonary and extra-pulmonary effects of vinpocetine. The pulmonary effects of VPN including inhibition of lung inflammation, ALI and ARDS through suppression of MAPK, NF-κB with release of NO. The extra-pulmonary effects of VPN including Improvement of brain neurotransmission and reduction cognitive dysfunction, inhibition of SARS-CoV-2-induced acute cardiac injury through inhibition of VGNC, inhibition of COVID-19-induced thrombosis by attenuating ED and platelets hyper-reactivity and inhibition of COVID-19-induced hyperviscosity by improvement of erythrocytes deformability. ALI: Acute lung injury; ARDS: Acute respiratory distress syndrome; ED: Erectile dysfunction; MAPK: Mitogen-activated protein kinase; NO: Nitric oxide; VGNC: Voltage gated Na^+2^ channel; VPN: Vinpocetine.

To date, there is no any of experimental or clinical trial studies or report regards the possible role of VPN in treating COVID-19. However, this brief report shed light on the crucial role of VPN in COVID-19 through PDE1 dependent and independent pathways. This drug is available and cheap with well-known safety profile and its use in the management of COVID-19 may open a new window for single drug with multiple effects. To support the role of VPN in COVID-19, *in silico* and *in vitro* preliminary studies are required to be an initial first step to confirm its role.

## Conclusion

VPN has noteworthy anti-inflammatory and anti-oxidant effects through inhibition of NF-κB/MAPK signaling pathway so, it may reduce SARS-CoV-2-induced hyper inflammatory and OS. As well, VPN has pulmonary and extra-pulmonary protective effects against COVID-19 through mitigation of OS, hyperinflammation, hyperviscosity, platelets hyper-reactivity and thrombosis. Clinical trials and prospective studies are mandatory in this concern to verify the beneficial effect of VPN in COVID-19.

## Future perspective

The authors are currently carrying out *in silico* and *in vitro* studies to confirm the possible effect of VPN on SARS-Cov-2 infection and hope to publish results of this new study in due course.

**Summary points**

- Vinpocetine (VPN) is a phosphodiesterase inhibitor, inhibits both basal and activated phosphodiesterase 1.
- VPN have anti-inflammatory effects through suppression the activity NF-κB signaling pathway and release of pro-inflammatory cytokines.
- VPN inhibits inflammatory signaling pathways including NLP3 inflammasome and MAPK that are triggered in SARS-CoV-2 infection.
- VPN has pulmonary and extrapulmonary protective effects against COVID-19 through mitigation of oxidative stress, hyperinflammation, hyperviscosity, platelets hyper-reactivity and thrombosis.
